# Performance of POCUS for Pregnancy Evaluation using a Non-Piezoelectric Ultrasound Device in the Emergency Department

**DOI:** 10.24908/pocusj.v10i02.18473

**Published:** 2025-11-17

**Authors:** Hae I. Yun, Brandon M. Wubben

**Affiliations:** 1 Carver College of Medicine, University of Iowa, 200 Hawkins Dr., Iowa City, IA, USA; 2 Department of Emergency Medicine, University of Iowa, 200 Hawkins Dr., Iowa City, IA, USA

**Keywords:** POCUS, Emergency Department, Handheld ultrasound, Obstetrics

## Abstract

**Background:**

Confirmation of pregnancy location using point of care ultrasound (POCUS) is an essential component of emergency obstetric care. The accuracy of a non-piezoelectric portable ultrasound device compared to obstetric sonographer-performed ultrasonography remains uncertain.

**Objective:**

The objective of this study was to describe the practical utility of a non-piezoelectric POCUS system (Butterfly iQ+) in the emergency department, including characterizing the accuracy of findings relative to obstetric sonographer-performed ultrasound (OB-US) for obstetric and non-pregnant pelvis evaluations in the emergency department.

**Methods:**

This retrospective cohort study was conducted at a Level 1 trauma center. Patients who underwent emergency physician-performed transabdominal obstetric or gynecologic POCUS examination using a non-piezoelectric transducer between November 2021 and November 2022 were included. Data from electronic medical records and ultrasound databases were abstracted. POCUS findings such as yolk sac presence, fetal heart motion, and gestational age were recorded alongside patient data including age, body mass index, human chorionic gonadotropin (hCG) levels, and ultrasound results. Descriptive statistics were analyzed using SPSS.

**Results:**

Seventy-two obstetric POCUS studies were included with an average maternal age of 29.6 ± 7.8 years. Common indications included positive pregnancy tests, abdominal/pelvic pain, and vaginal bleeding. All first-trimester intrauterine pregnancies (IUPs) diagnosed by POCUS and referred for OB-US were confirmed, with no false positives. POCUS had a sensitivity of 83.3% (95% CI 61.8-94.5) and specificity of 100% (95% CI 71.7-100) for confirmation of an IUP. The lowest hCG levels at which IUP was detected with POCUS were 6,488 without fetal heart motion and 7,098 with heart motion. The agreement between POCUS and OB-US for gestational age measurements was moderate (ICC = 0.83, 95% CI 0.0-0.99). In the second trimester, POCUS accurately detected fetal heart rate, with a strong agreement for gestational age (ICC = 0.96, 95% CI 0.43-0.99) compared to OB-US. Transabdominal POCUS also identified gynecologic conditions such as postpartum hemorrhage, and normal POCUS exams led to alternate diagnoses such as pelvic inflammatory disease and pyelonephritis.

**Conclusion:**

POCUS using a non-piezoelectric ultrasound device was able to accurately rule in first trimester IUP in the emergency department at relatively low hCG levels, with no false positive IUPs identified. During the second and third trimesters, POCUS consistently detected fetal heart motion and had good accuracy for gestational age measurements compared to OB-US. These findings suggest that POCUS using a non-piezoelectric ultrasound device is a useful tool for emergency department assessments, though further evaluation is needed.

## Introduction

Confirmation of first-trimester pregnancy location using ultrasound is an essential component of emergency obstetric care. Point of care ultrasound (POCUS) has previously been shown to facilitate faster diagnosis and treatment in cases of ruptured ectopic pregnancy [1,2]. POCUS in the emergency setting has traditionally been performed using cart-based systems. However, newer handheld ultrasound devices, including those using non-piezoelectric technology, are now available and gaining popularity [3]. Non-piezoelectric ultrasound devices, such as the Butterfly iQ+ (Butterfly Network Inc., Burlington MA), which use a capacitive micromachined array to emulate a piezoelectric array, have demonstrated reasonable specificity for other emergent diagnoses, such as ruling in pericardial effusion, pleural effusion, and deep vein thrombosis [4].

Some handheld ultrasound devices have been shown to perform well in an obstetric clinic setting, demonstrating good agreement with traditional sonography for fetal measurements such as crown-rump length [5,6]. Handheld devices have demonstrated high accuracy and reliability in detecting intrauterine pregnancies (IUPs) compared to comprehensive transabdominal ultrasound, with excellent inter-rater agreement and consistent gestational age assessment [6–8]. One prior abstract prospectively compared Butterfly iQ to a cart-based system which were both used by emergency providers in a convenience sample of patients. It described a diagnostic accuracy of 96% (95% CI 85-100%) for confirming an IUP in an emergency department setting. However, two cases of IUP were missed, as they were seen only with the cart-based system, and there was no comparison to obstetric sonographer-performed imaging [9].

Although POCUS has performed well in an obstetric clinic setting, there is currently limited evidence that it is accurate for detecting IUP in the emergency department, and the performance of POCUS in the emergency department relative to sonographer-performed ultrasound is unknown. Additionally, although the discriminatory zone for pregnancy detection using cart-based devices has been well-studied, the beta-human chorionic gonadotropin (hCG) detection threshold for newer, non-piezoelectric ultrasound devices is also unknown [7]. Therefore, the objective of this study was to describe the utility of a non-piezoelectric ultrasound system (Butterfly iQ+) in the emergency department, including accuracy relative to obstetric sonographer-performed ultrasound (OB-US) for pregnancy and non-pregnant pelvis evaluations in the emergency department, and reporting the beta-hCG levels at which an IUP could be detected.

## Methods

We conducted a retrospective review of electronic medical records and a POCUS database at our Level 1 trauma center and emergency department, which has more than 50,000 annual patient visits. The site is an academic teaching hospital affiliated with a three-year emergency medicine residency program, an obstetrics and gynecology residency, and has readily available consultative sonography. First, our emergency ultrasound quality assurance database (Butterfly Network, Burlington, MA) was queried for patients who underwent transabdominal obstetric or gynecologic POCUS performed by emergency medicine residents or faculty in the emergency department, from November 2021 to November 2022, using a non-piezoelectric ultrasound system (Butterfly iQ+, Butterfly Network Inc., Burlington, MA). Second, standardized data abstraction was performed from the electronic medical record (EMR) and emergency ultrasound quality assurance database using a standardized form hosted on REDCap. All scans undergo quality assurance by fellowship-trained emergency medicine ultrasound faculty.

A second reviewer independently verified over 10% of the abstracted EMR records for accuracy. Recorded POCUS findings included the presence or absence of a yok sac and gestational age measurements. The presence of an IUP was defined as the presence of a gestational sac with yolk sac, regardless of fetal heart motion. Patient-specific variables included age, body mass index (BMI), beta-hCG results (quantitative or qualitative), and the results from obstetric sonographer-performed transabdominal or transvaginal ultrasound. SPSS (IBM SPSS Statistics for Macintosh, Version 28.0, Armonk, NY) was used for statistical analysis. The sensitivity, specificity, negative predictive value, and positive predictive value were calculated. This study was granted a waiver of written informed consent by our institutional review board.

## Results

There were 72 POCUS examinations performed during the study period using a non-piezoelectric ultrasound device. Of these, 58 were transabdominal obstetric POCUS scans; one was a twin gestation and the remainder singleton, with an average maternal age of 28.2 ± 6.2 years and an average body mass index (BMI) of 28.2 ± 8.6 kg/m2. The most common indications for POCUS included patient-reported positive pregnancy (63%), abdominal or pelvic pain (47%), and/or vaginal bleeding (26%). The most common type of POCUS evaluation was for confirmation of pregnancy or pregnancy viability under 13 weeks of gestation (37/72, 51%). Although emergency medicine residents participated in most scans, the primary author of the POCUS documentation was emergency medicine faculty in 39% of cases, a third-year resident in 24% of cases, a second-year resident in 19% of cases, and an intern in 18% of cases.

For the 37 patients presenting in the first trimester, POCUS diagnosed an IUP in 20 cases and did not identify an IUP in 17 cases (including one ectopic pregnancy). There were 20 first-trimester POCUS scans referred for an OB-US, of which 16 (80%) had transvaginal ultrasound performed, and the remainder had transabdominal ultrasound (Table 1). There were 17 POCUS studies that were not sent for consultative ultrasound. All POCUS diagnoses of IUP that were referred for an OB-US were correct based on the OB-US, although heart motion detection was variable (Table 2). Gestational age measurements for the four patients who had both POCUS and OB-US had a moderate agreement (ICC = 0.83, [95% CI 0-.99]), with the lower bound markedly affected by an estimate for the twin gestation that was more than two weeks below the OB-US estimate in the setting of a small sample size. The lowest hCG level at which an IUP was detected with POCUS was 6,488 mIU/mL without fetal heart motion and 7,092 mIU/mL with heart motion.

**Table 1.** Comparison of findings between first-trimester emergency physician-performed obstetric point of care ultrasound (POCUS) using a non-piezoelectric ultrasound device and obstetric sonographer-performed ultrasound (OB-US) for cases referred for consultative ultrasound.

Comparison	Any OB-US		Transabdominal OB-US		Transvaginal OB-US	
**Finding**	POCUS	Any OB-US	POCUS	Transabdominal OB-US	POCUS	Transvaginal OB-US
**Yolk sac**	7/20 (35%)	11/20 (55%)	3/4 (75%)	3/4 (75%)	4/16 (25%)	8/16 (50%)
**Fetal pole**	6/20 (30%)	9/20 (45%)	2/4 (50%)	3/4 (75%)	4/16 (25%)	6/16 (38%)
**Fetal heart motion**	5/20 (25%)	7/20 (35%)	2/4 (50%)	3/4 (75%)	3/16 (19%)	4/16 (25%)
**Free pelvic fluid**	0/20 (0%)	5/20 (25%)	0/4 (0%)	0/4 (0%)	0/16 (0%)	5/16 (31%)

**Table 2.** Comparison of emergency physician-performed first-trimester obstetric point of care ultrasound (POCUS) using a non-piezoelectric ultrasound device versus sonographer-performed obstetric ultrasound (OB-US) diagnoses for cases referred for consultative ultrasound (n = 20).

POCUS Diagnosis		OB-US Diagnosis
IUP with heart motion	5/20 (25%)	IUP with HM= 4
		IUP without HM = 1
IUP without heart motion	2/20 (10%)	IUP without HM = 1
		IUP w/=ith HM = 1
No definite IUP	13/20 (65%)	No IUP = 8
		IUP with HM = 2
		IUP without HM = 2
		Ectopic = 1
Visualized ectopic	0/20 (0%)	N/A

IUP = intrauterine pregnancy; HM = heart motion.

Of those diagnosed with an IUP, 19 out of 20 were able to be confirmed as true positives by either consultative OB-US (n = 7) or clinical follow-up (n = 12), with the remaining patient lost to follow-up. The overall performance of obstetric POCUS relative to OB-US or clinical follow-up for first-trimester IUP confirmation yielded a sensitivity of 82.6% (95% CI 60.5-94.3) and specificity of 100% (95% CI 71.7-100). There were no known IUP false positives using POCUS and only four cases of IUP which were not identified with POCUS that were identified with transvaginal OB-US.

There were 19 POCUS evaluations in the second trimester; the majority (68%) were not referred for consultative OB-US. POCUS accurately detected fetal heart motion in 5 out of 5 cases (100%) referred for consultative transabdominal OB-US, and had excellent agreement for gestational age measurements (ICC = 0.96 [95% CI 0.43-0.99]). Gestational age in the second trimester was measured variably, typically including biparietal diameter, but dependent on sonographer practice. There were only two cases of third-trimester pregnancy that presented to the emergency department for traumatic injuries. In both cases, a fetal heart rate was present but there were no other significant findings noted, both without consultative imaging for comparison.

There were 14 transabdominal gynecologic POCUS scans performed for evaluation of the non-pregnant pelvis, with an average patient age of 30.5 ± 13.3 years and BMI of 21.2 ± 1.2 kg/m2. The most common indications for POCUS were abdominal and pelvic pain (71%) or vaginal bleeding (29%). Although there was a wide range of ultimate diagnoses, POCUS was used without referral for consultative OB-US for identification of an empty uterus in the setting of postpartum hemorrhage, and for several normal exams that ultimately received other diagnoses such as pelvic inflammatory disease without tubo-ovarian abscess and pyelonephritis. For the seven cases (50%) referred for consultative OB-US, notable findings that may have prompted additional imaging were free pelvic fluid and multiple ovarian cysts, with final diagnoses including ovarian hyperstimulation syndrome and a large ovarian cyst that was over 20 cm in size.

## Discussion

We found that POCUS using a non-piezoelectric ultrasound device was accurate in detecting an IUP, including at relatively low beta-hCG levels. This suggested its possible utility in the emergency department for timely diagnosis of an IUP, especially in early pregnancy where excluding ectopic pregnancy is essential.

Prior studies examining obstetric POCUS in the emergency department setting have shown mixed results, including good agreement with cart-based POCUS systems. However, there are certain limitations in visualizing complex pelvic structures and ectopic pregnancies compared to OB-US [5,10,11]. Our study aligns with prior findings that POCUS using a non-piezoelectric ultrasound device can be useful for initial assessments in obstetric and gynecologic emergencies but does not obviate the need for consultative ultrasound in all clinical contexts.

In our study, for non-pregnant pelvic evaluations, POCUS identified several significant clinical findings that were able to be confirmed with consultative OB-US. Several normal gynecologic POCUS exams further led to alternate diagnoses. Importantly, abnormal transabdominal gynecologic POCUS findings frequently and appropriately prompted additional consultative imaging, such as free pelvic fluid and multiple ovarian cysts, which led to a diagnosis of ovarian hyperstimulation syndrome. Such findings highlight the potential of POCUS as a valuable adjunct to clinical assessment in the emergency department. However, POCUS likely has limitations in detecting and characterizing more complex pelvic abnormalities such as ovarian torsion and ovarian cysts, necessitating further evaluation with traditional imaging modalities. A prior study comparing POCUS with a high-end system across three patient categories reported good to very good agreement in identifying key obstetric and gynecological parameters (endometrial thickness, ovarian mass mean diameter) [5]. Overall, POCUS may be sufficiently accurate to triage patients with bleeding or pain, in addition to assessing patients for the presence or absence of large pelvic masses in the evaluation of gynecological pathology. Additionally, if POCUS fails to detect an IUP, this can assist in the triage process, advocating for prompt follow-up with obstetric transvaginal ultrasound or surgical consultation if clinically appropriate. However, POCUS using a non-piezoelectric ultrasound should not be viewed as a replacement for cart-based POCUS or OB-US, particularly in medical centers with readily available obstetric sonography. Rather, we postulate that the highest value of POCUS using a non-piezoelectric ultrasound device may be in its utility in resource-limited settings, where access to OB-US is limited or absent and early referral or transfer may be lifesaving.

## Limitations

Several limitations should be considered when interpreting the findings of this study. Because it was retrospective, it is possible that some patients received undocumented POCUS that was not included in the study. We were unable to determine why consultative ultrasound was or was not obtained. The limited sample size of the study could potentially restrict the applicability of the findings to a wider population. The agreement between POCUS and OB-US for gestational age measurements is based on a relatively small sample, introducing a degree of uncertainty. Additionally, the patients' awareness of their pregnancy status, along with the possibility of alternative imaging modalities being obtained before their arrival, could introduce bias into the interpretation of the POCUS results. A significant limitation of this study was the exclusive use of transabdominal POCUS. Transvaginal sonography is often necessary to rule out ectopic pregnancy, ovarian torsion, and to identify subtle gynecologic pathology, but requires a specialized endocavitary transducer that was not available for the POCUS system during the study period. Finally, this study used only the Butterfly iQ+ device, and findings may not be applicable to other POCUS systems. Future research with larger, prospective studies is needed to further validate the findings and explore the comparative effectiveness of POCUS versus traditional imaging modalities in various clinical scenarios.

## Conclusion

POCUS using a non-piezoelectric ultrasound device was able to accurately rule in first trimester IUP in the emergency department at relatively low hCG levels, with no false positive IUPs identified. During the second and third trimesters, POCUS demonstrated consistent detection of fetal heart motion and had good accuracy for gestational age measurements compared to OB-US. While more studies with larger sample sizes and a wider variety of clinical scenarios are warranted, the findings suggest that POCUS could be a useful tool for initial obstetrics and gynecology-related assessments.
